# Unveiling the genetic landscape and motility-associated functions of Type IV pili in the *Bifidobacterium* genus

**DOI:** 10.20517/mrr.2025.119

**Published:** 2026-03-19

**Authors:** Gabriele Andrea Lugli, Emanuele Selleri, Chiara Tarracchini, Aryanna Muscò, Silvia Petraro, Giulia Longhi, Leonardo Mancabelli, Christian Milani, Francesca Turroni, Marco Ventura

**Affiliations:** ^1^Laboratory of Probiogenomics, Department of Chemistry, Life Sciences, and Environmental Sustainability, University of Parma, Parma 43124, Italy.; ^2^Microbiome Research Hub, University of Parma, Parma 43124, Italy.; ^3^Department of Medicine and Surgery, University of Parma, Parma 43124, Italy.

**Keywords:** Bifidobacteria, genomics, phylogenetics, extracellular structures, T4P

## Abstract

**Background:** Bifidobacteria are recognized as one of the most influential bacterial groups inhabiting the human gut, capable of modulating the host’s health. To understand how these bacteria interact with their hosts, it is essential to investigate their extracellular structures, such as pili. While the presence of sortase-dependent pili has already been investigated in the *Bifidobacterium* genus, limited information is available on Type IV pili (T4P), which have previously been identified in a few species as tight adherence (Tad) loci.

**Methods: **Here, we explored the T4P distribution across the currently 117 (sub)species representing all described taxa of the *Bifidobacterium* genus, revealing two distinct loci unevenly distributed within the genus through *in silico* genomic analyses supported by *in vitro* validation.

**Results: **Our analysis identified a conserved Type IVc pili (T4cP) structure across all bifidobacterial taxa, with minor predicted structural variations in members of the *Bifidobacterium longum* and *Bifidobacterium boum* phylogenetic groups. This T4cP structure, also known as Tad, exhibited an ancestral, non-retractile architecture typically associated with stable colonization and long-term persistence. In addition, a secondary Type IVa pili (T4aP) structure was detected in 13 bifidobacterial species. These species are associated with specific ecological niches, including primate, bovine, and porcine hosts, suggesting a link between this locus and host-associated adaptation.

**Conclusion: **Notably, twitching motility assays demonstrated that *Bifidobacterium adolescentis* strains harboring the T4aP locus exhibit motility in response to specific environmental signals, observed upon starch supplementation of the growth medium, thereby challenging the traditional view of bifidobacteria as a strictly non-motile bacterial genus.

## INTRODUCTION

Bifidobacteria are a group of Gram-positive bacteria primarily inhabiting the gastrointestinal tract of mammals and social insects^[1,2]^. Only a few species have been identified in non-host-associated environments, representing exceptions to this typical host-adapted lifestyle^[3-5]^. However, these species can also be considered relevant to human health, as their peculiar ecological niche, i.e., fermented foods, makes them accessible to human consumption^[6-8]^. Thus, understanding how different species of bifidobacteria interact with the human host is a crucial step for uncovering the molecular mechanisms underlying their colonization and persistence in the human gut.

Bacteria expose many extracellular structures that can interact with the host. To name a few, pili, fimbriae, polysaccharide capsule, S-layer proteins, teichoic and lipoteichoic acids, exopolysaccharides, adhesins, secretion systems, and outer membrane vesicles are all structures produced by microorganisms that mediate adhesion, host cell recognition, and immune modulation^[9-13]^. Among them, pili show a rich variety of structures, such as Type I Pili (Chaperone-Usher Pathway Pili), Type III Secretion-Associated Pili (Injectisome Needle), Type IV Pili (T4P), Type V Pili (Curli Fibers), Type VI Pili (Contractile Injection System), Sortase-Dependent Pili, Conjugative Pili, and Competence Pili^[14-16]^. The presence of sortase-dependent pili has already been discussed in bifidobacteria, revealing considerable genetic variability of these structures among bifidobacterial species, which appears to have been subjected to horizontal gene transfer (HGT) events^[17-19]^. On the other hand, the T4P encoded by the Tad (tight adherence) locus has been previously identified and characterized in the genome of *Bifidobacterium breve* (*B. breve*) UCC2003^[20,21]^, and *Bifidobacterium animalis *(*B. animalis*) subsp. *lactis* A6^[22]^ and CNCM I-4602^[23]^.

T4P are dynamic filamentous appendages found in many bacteria, playing key roles in motility, adhesion, DNA uptake, and host interaction^[24]^. Based on sequence and structural differences, T4P have been traditionally classified into two major groups, including the type IVa pili (T4aP) and type IVb pili (T4bP), each with distinct components and functional specializations^[25]^. Furthermore, the Tad locus was first identified in *Aggregatibacter actinomycetemcomitans* as essential for biofilm formation and strong surface attachment^[26]^. Although historically considered a variant of type IV pili, phylogenomic analyses have recently reclassified Tad systems as a distinct subgroup, now referred to as Type IVc pili (T4cP)^[27]^. This classification reflects their unique evolutionary origin and structural features, setting them apart from other type IV pili systems.

In this study, we explore the biodiversity of the T4P across all members of the genus *Bifidobacterium*, aiming to understand from a genomic and phylogenetic perspective how this complex structure has evolved during the species differentiation of these bacteria. Accordingly, we have collected all RefSeq genomes of the species from the National Center for Biotechnology Information (NCBI) database (https://www.ncbi.nlm.nih.gov/datasets/genome/), encompassing 2,665 high-quality genomes in which we have mined the genetic signature of T4P loci. Additionally, *in vitro* experiments were performed to corroborate the *in silico* data, highlighting a movement of *Bifidobacterium adolescentis* (*B. adolescentis*) strains that encompass the T4P loci in their genomes.

## METHODS

### Bifidobacterial genome sequence selection

Complete and partial genome sequences of *Bifidobacterium* strains were retrieved from the RefSeq NCBI genome database (https://www.ncbi.nlm.nih.gov/datasets/genome/)^[28]^, representing the most up-to-date collection of publicly available genome sequences of this taxon, encompassing 117 subspecies [Supplementary Table 1]. All 2,665 RefSeq genomes were analyzed to investigate the intraspecific variability of the T4P loci identified within the framework of the project [Supplementary Table 2]. Genome sequences included in this study were validated employing fastANI [Average Nucleotide Identity (ANI) > 95%] ^[29]^ and CheckM2 (completeness > 95% and contamination < 5%) ^[30]^. 

### Gene prediction and annotation

To avoid discrepancies in gene prediction across the different species of the genus *Bifidobacterium*, the protein-coding sequences of each strain analyzed in the framework of this study were managed by the MEGAnnotator2 pipeline^[31]^ using the gene-finding program Prodigal^[32]^. Predicted open reading frames (ORFs) were functionally annotated by means of the software Diamond^[33]^ performed against the whole NCBI-nr database resized with a Cluster Database at High Identity with Tolerance (CD-HIT) sequence identity threshold of 70%^[34]^, coupled with hidden Markov model proﬁle (HMM) searches using InterProScan^[35]^ performed against the manually curated Pfam-A database (https://ftp.ebi.ac.uk/pub/databases/Pfam/)^[36]^.

### Bifidobacterial Type IV Pili database

The predicted protein-coding sequences of bifidobacteria were screened to identify genes encoding Type IV Pili based on sequence similarity to genes classified in the RefSeq NCBI database (https://www.ncbi.nlm.nih.gov/refseq/)^[28]^. A first screening was performed against 117 reference genomes covering all subspecies of the genus *Bifidobacterium* to explore the genome variability between species [Supplementary Table 1]. The collection of the resulting genes enabled the generation of the *Bifidobacterium* Type IV Pili database, constituted by 1,042 genes predicted to encode the loci variability within the genus [Supplementary Table 2]. Then, by using the *Bifidobacterium* Type IV Pili database, a second screening was performed against the protein-coding sequences of each bifidobacterial genome retrieved from the RefSeq NCBI genome database (https://www.ncbi.nlm.nih.gov/datasets/genome/), aiming to identify orthologs within each species. The identified loci were then visualized as genetic maps using a custom Python script.

### Comparative genomics

Predicted protein-coding sequences derived from each genome were used for pangenome reconstruction using the software Roary^[37]^. In detail, orthologous sequences were identified through an all-against-all comparison using Protein Basic Local Alignment Search Tool (BLASTp) with an 80% sequence identity and then organized into functional clusters of orthologous groups through the MCL algorithm (graph-based Markov clustering algorithm). Functional annotation of clusters of orthologous genes (COGs) was attributed by using the MEGAnnotator2 pipeline^[31]^.

### Phylogenetic analysis of the genus *Bifidobacterium*

Core genes, i.e., protein families shared between all genomes, were defined by selecting families containing at least one single protein member for each genome. The concatenated core genome sequences were aligned using MAFFT (Multiple Alignment using Fast Fourier Transform)^[38]^, and the corresponding phylogenomic tree was constructed using the neighbor-joining method in ClustalW version 2.1^[39]^. The core genome tree was built using Interactive Tree of Life (iTOL) v7^[40]^.

### Identification of putative HGT events

Bifidobacterial gene sequences were analyzed based on codon usage bias (CUB) and guanine-cytosine content (GC) content. Three metrics were used to assess CUB: Relative Synonymous Codon Usage (RSCU), Effective Number of Codons (ENC), and the Codon Adaptation Index (CAI). The thresholds of all four metrics, including GC content, were dynamically calculated by measuring deviation from the values observed in the other coding sequences within the same genome. For each metric, a gene was considered TRUE or FALSE depending on whether its value deviated from at least 90% of the other genes in the genome. Specifically, values above the 90th percentile for GC content, RSCU, and ENC, and below the 10th percentile for CAI.

### Strains and culture conditions

Bifidobacterial strains used in this study were *B. adolescentis* ATCC 15703^T^ (type strain obtained from American Type Culture Collection), *B. adolescentis* 1892B, *B. adolescentis* 2301B, and *B. adolescentis* PRL2014^[41]^. *B. adolescentis* was selected as a model species for phenotypic analyses because genomic screening predicted the presence of the T4aP locus in this species, with detectable strain-level variability allowing comparison between strains harboring or lacking the locus. Each bacterium was initially revived from glycerol stocks and cultivated overnight, anaerobically (2.99% H_2_, 17.01% CO_2_, and 80% N_2_) (Concept 400; Ruskin) at 37 °C, in de Man-Rogosa-Sharpe (MRS) broth (Sharlau Chemie, Barcelona, Spain) supplemented with 0.05% (wt/vol) L-cysteine-HCl.

### Twitching motility assay

Twitching motility was analyzed by different protocols using the agar stab methods^[42,43]^. All bifidobacterial strains were grown at 37 °C on 0.5% MRS agar and 0.5% MRS agar added with 2% (w/v) starch, anaerobically (2.99% H_2_, 17.01% CO_2_, and 80% N_2_). Briefly, plates with 25 mL were prepared a day before the assays and allowed to sit on the benchtop overnight. These plates were then dried in a biosafety cabinet for 20 min before stab inoculation. After 96 h of incubation, the agar media were removed, and the twitching zone was visualized.

## RESULTS AND DISCUSSION

### Identification of the Type IV Pili of Bifidobacteria

T4P are multifunctional surface structures playing a crucial role in bacterial processes, including adhesion, motility, and host interaction. Notably, in bifidobacteria, they have been shown to play a relevant role in establishing a stable colonization in the gastrointestinal tract^[20,21]^. Thus, to fully explore the T4P genetic biodiversity within the genus *Bifidobacterium*, 117 reference strains of this genus, representing the whole (sub)species biodiversity of the taxon, were included in this screening [Supplementary Table 1].

**Figure 1:**
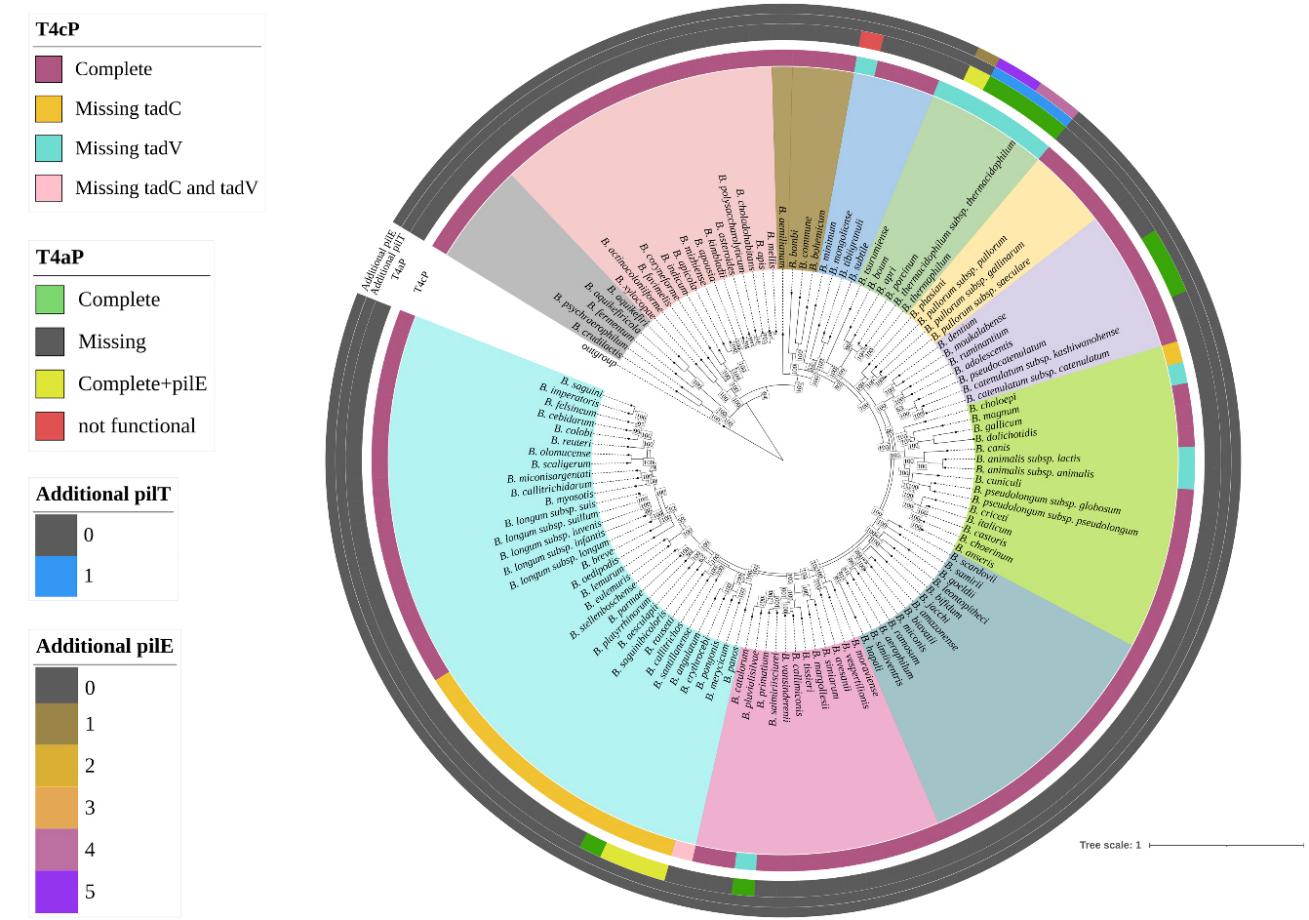
Distribution of T4P structure across the *Bifidobacterium* genus. The phylogenomic tree is based on the concatenation of 174 core protein sequences of 117 reference strains of the genus. The tree was constructed by the Neighbour-joining method, with the genome sequence of *Scardovia inopinata* JCM 12537 as outgroup. The different colors in the inner circle indicate the division into phylogenetic groups, while the outer circles illustrate the distribution and configuration of the T4P. T4P: Type IV pili; T4aP: type IVa pili; tad: tight adherence.

Each analyzed *Bifidobacterium* genome exhibited at least one or two extracellular structures, as indicated by the presence of genes homologous to those associated within T4P systems in other microorganisms [Figure 1]. Consistent with the organization of multiprotein extracellular structures such as pili, the two identified T4P structures were located in distinct loci across the bifidobacterial chromosomes, forming gene clusters likely transcribed as operons. Notably, a conserved structure was identified in each subspecies of the genus, revealing the presence of a core extracellular machinery employed by bifidobacteria to interact with their environment. This first T4P cluster had been previously identified and classified as Tad pilus, also referred to as T4cP, in *B. breve* UCC2003^[20]^ and two *B. animalis* strains^[22,23]^ [Supplementary Table 2]. In addition, a second T4P structure was identified in 13 reference bifidobacterial strains, revealing a locus composition resembling that of Type IVa pili (T4aP) found in other Gram-positive bacteria^[44]^ [Supplementary Table 2].

To better understand the genetic composition of both structures across the *Bifidobacterium* genus, an in-depth correlation analysis was performed in relation to the phylogeny of the entire taxon. The identification of a conserved T4cP locus across all subspecies suggests the presence of a stable, genus-wide extracellular machinery likely involved in fundamental ecological functions. In contrast, the more restricted distribution of the T4aP locus raises the possibility of niche-specific adaptation and functional diversification within selected bifidobacterial taxa.

### The Tad locus is an ancestral T4P extracellular structure of the genus *Bifidobacterium*

**Figure 2:**
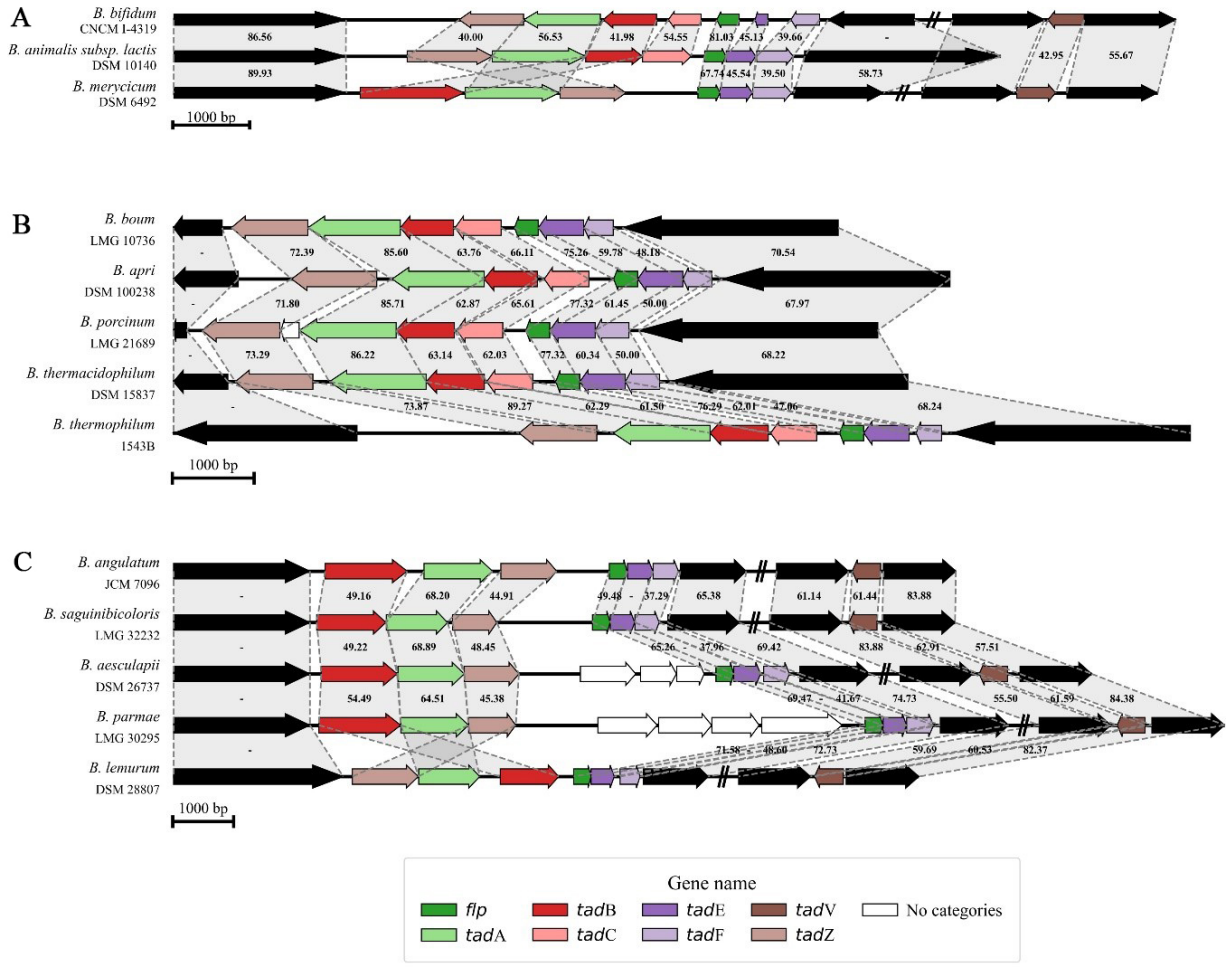
Genetic maps of the T4cP loci in bifidobacteria. (A) shows the genetic map of the three different configurations of the T4cP locus distributed across the genus *Bifidobacterium*; (B and C) display the degree of conservation of two T4cP configurations within the *B. boum* phylogenetic group and a *B. longum* sub-group, respectively. Each arrow indicates an ORF, whereas the length of the arrow is proportional to the length of the predicted ORF. Individual genes within the locus, i.e., *tad*Z, *tad*A, *tad*B, *tad*C, *flp*, *tad*E, *tad*F, and *tad*V, are distinguished by different colors, and identity values to the reference gene are shown between the genes. T4cP: Type IVc pili.* B. boum*: *Bifidobacterium boum*; ORF: open reading frame; tad: tight adherence; *B. longum*: *Bifidobacterium longum*.

Across all analyzed bifidobacterial chromosomes, eight genes associated with the assembly, secretion, and biogenesis of the T4cP were identified, i.e., *tad*Z, *tad*A, *tad*B, *tad*C, *flp*, *tad*E, *tad*F, and *tad*V [Figure 2]. Within the genus, two distinct genetic constellations of the T4cP locus were revealed, each featuring two associated gene clusters encompassing six to seven of the genes listed above. More in detail, using *Bifidobacterium bifidum* (*B. bifidum*) as a reference strain, the first of these clusters was predicted to include genes *tad*Z, *tad*A, *tad*B, and *tad*C, which are involved in pilus localization, assembly, and export [Figure 2]. The second cluster, placed directly in tandem with the former, is predicted to comprise the *flp* gene encoding the prepilin and the two *tad*E and *tad*F genes encoding the two pseudopilins [Figure 2]. A second, less common pattern of the T4cP locus was identified in 16 bifidobacterial species [Figure 1], characterized by the absence of the *tad*C gene and an inversion of the gene cluster predicted to encode for the prepilin and pseudopilin [Figure 2]. Nevertheless, both *tad*B and *tad*C genes were predicted to encode integral membrane proteins (or inner membrane core proteins) that form the complex responsible for the pilus anchoring to the bacterial membrane. Therefore, given their overlapping predicted function and the presence of this configuration in 16 different species, the absence of the *tad*C gene may not necessarily impair pilus export functionality.

Despite the highly conserved nature of the locus, the gene *tad*V encoding the prepilin peptidase protein, when present, was consistently placed outside the T4cP genetic cluster, corroborating its first classification in *B. breve* UCC2003^[20]^. Interestingly, a homologous *tad*V gene encoding the peptidase was not identified in 12 bifidobacterial species [Figure 1].

To investigate the distribution of the T4cP locus from an ecological perspective, a pangenome analysis involving the 117 reference strains allowed the selection of 174 core genes whose sequences were used to reconstruct the most up-to-date bifidobacterial phylogeny [Figure 1]. Interestingly, the distribution of the T4cP configuration lacking the *tad*C gene showed a strong phylogenetic correlation within the genus. Specifically, 15 out of 16 species missing the gene clustered within a subgroup of the *Bifidobacterium longum* (*B. longum*) phylogenetic group, comprising species typically associated with the gut microbiota of primates [Figure 1]. Similarly, the absence of the *tad*V gene also appears to be linked to the evolutionary history of the *Bifidobacterium* genus. While six species lacking this gene were dispersed across four phylogenetic groups, all six species belonging to the *Bifidobacterium boum* (*B. boum*) phylogenetic group consistently lacked *tad*V [Figure 1]. To further validate our findings, 2,665 RefSeq genomes belonging to the *Bifidobacterium* genus were downloaded from the NCBI database (https://www.ncbi.nlm.nih.gov/datasets/genome/) [Supplementary Table 3], allowing us to investigate the intraspecies variability revealing a high conservation of the T4cP within species [Supplementary Table 4].

Altogether, our *in silico* screening highlighted a conserved T4cP architecture across bifidobacterial species, with only minor genetic variations associated with the absence of the *tad*C or *tad*V gene. The overall conservation of this locus, along with its presence in species representing early-diverging phylogenetic groups of the genus - including those associated with social insects and fermented milk^[2,3,5]^ [Figure 1] - supports the notion that T4cP is an ancestral, vertically inherited extracellular structure. The predicted non-retractile nature of this structure further suggests a primary role in stable surface attachment and long-term ecological persistence rather than dynamic motility^[45,46]^. The phylogenetic clustering of specific locus variants, such as the recurrent absence of *tadC* within the *B. longum* group and *tadV* within the *B. boum* group, indicates that subsequent lineage-specific refinements may have occurred without disrupting the core functionality of the system. 

### The T4aP locus encodes a structure that may confer motility to bifidobacteria

**Figure 3:**
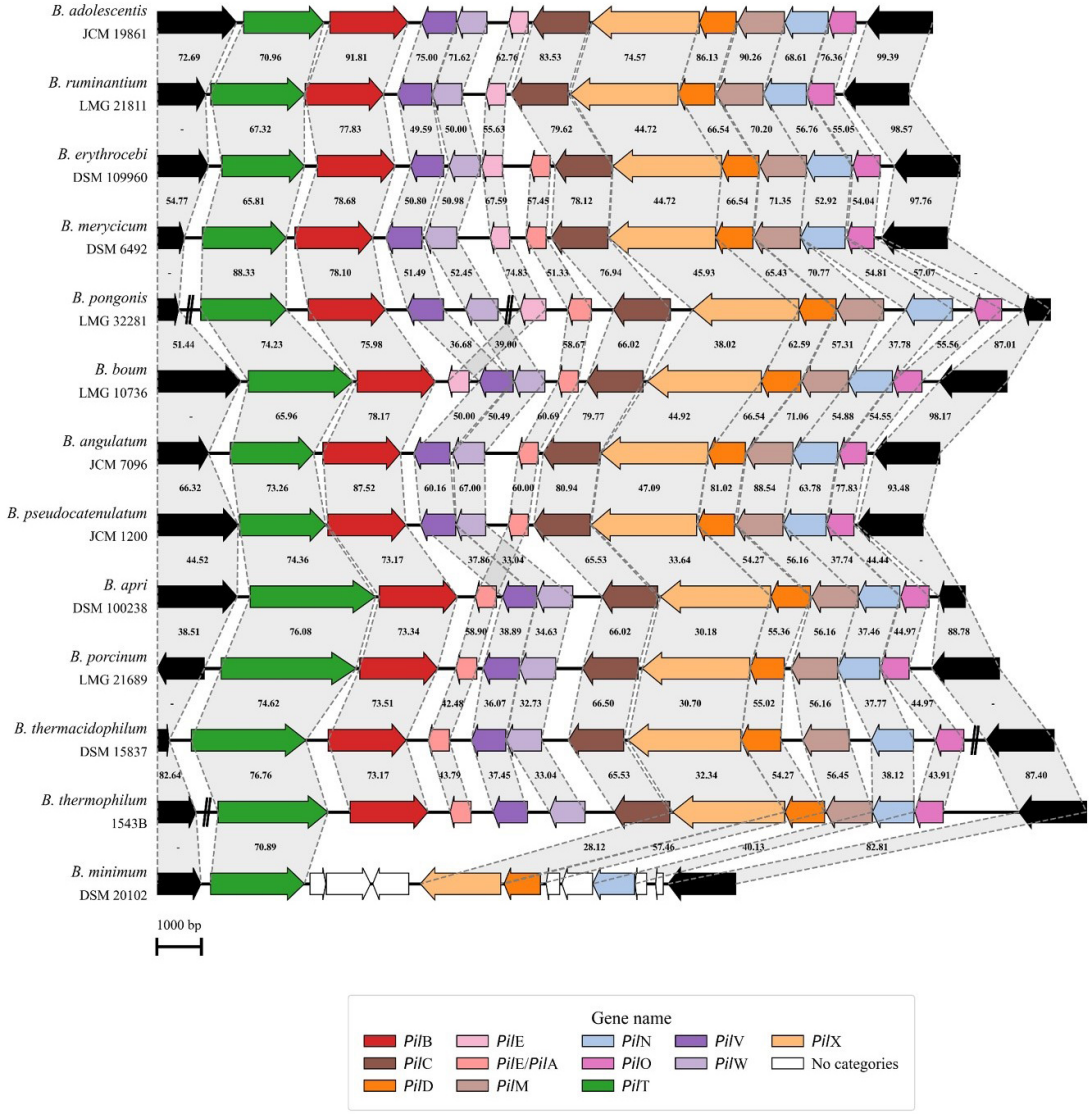
Genetic map of the T4aP locus in bifidobacteria. Representation of the genetic organization of the T4aP locus across 13 *Bifidobacterium* species. Each arrow indicates an ORF, whereas the length of the arrow is proportional to the length of the predicted ORF. Individual genes within the locus, i.e., *pil*T, *pil*B, *pil*V, *pil*W, *pil*E/*pil*A, *pil*C, *pil*X, *pil*D, *pil*M, *pil*N, and *pil*O, are distinguished by different colors, and identity values to the reference gene are shown between the genes. T4aP: Type IVa pili; ORF: open reading frame.

Unlike the previously described conserved T4cP structure, only 13 of the 117 reference strain genomes exhibited a secondary T4P structure, which appeared to be a more complex pilus resembling a T4aP. Using the nomenclature based on *Clostridium perfringens*^[44]^ and other Gram-positive bacteria in which this structure has already been characterized, eleven genes were functionally classified to belong to the locus, i.e., *pil*T, *pil*B, *pil*V, *pil*W, *pil*E/*pil*A, *pil*C, *pil*X, *pil*D, *pil*M, *pil*N, and *pil*O [Figure 3 and Supplementary Table 1].

The locus structure starts with two genes predicted to be involved in pilus assembly and retraction, i.e., *pil*B and *pil*T. Then, on the opposite DNA strand, nine additional genes are present, starting with *pil*E/*pil*A, encoding the prepilin, and *pil*V, *pil*W, and *pil*X, encoding pseudopilins [Figure 3]. Following is the *pil*C gene, encoding an inner membrane core protein, *pil*D, encoding a prepilin peptidase, and three inner membrane accessory genes *pil*M, *pil*N, and *pil*O. What makes this T4P structure particularly interesting compared to the more conserved T4cP described above is the presence of the *pilT* gene, which is a hallmark of dynamic pili capable of retraction by disassembling pilin subunits from the pilus structure. The identification of this structure suggests that a few *Bifidobacterium* species possess a more evolved T4P system, capable of supporting more complex cellular dynamics.

The T4aP locus showed a consistent genetic organization across the 13 identified species, except for *Bifidobacterium minimum*, where genetic rearrangement, gene loss, and frameshifts revealed a non-functional structure [Figure 3]. Moreover, in the genomes of five species - *B. boum, Bifidobacterium erythrocebi, Bifidobacterium merycicum, Bifidobacterium pongonis,* and *Bifidobacterium ruminantium* - an extra *pilE* was identified within the locus, while additional prepilin genes were found scattered throughout the genomes of members of the *B. boum* phylogenetic group [Figure 1]. Interestingly, members of the *B. boum* phylogenetic group exhibited the most distinctive Type IV configurations, characterized by the absence of the *tad*V gene associated with T4cP and the presence of additional prepilin genes, which may contribute to a structurally diverse T4aP pilus composition [Figure 1]. As observed for the T4cP locus, the screening of 2,665 RefSeq *Bifidobacterium* genomes revealed a high level of conservation of the T4aP locus within each species in which it was identified [Supplementary Table 4].

Interestingly, from an ecological perspective, the species carrying the T4aP locus in their genomes were distributed across the *B. boum*, *B. longum*, and *B. adolescentis* phylogenetic groups, which are primarily associated with primate, bovine, and porcine hosts^[47-50]^. Among the 12 species predicted to encode a functional structure, *B. adolescentis* and *Bifidobacterium pseudocatenulatum* (*B. pseudocatenulatum*) stand out as particularly interesting, as they were predominantly isolated from the human gut and represent the only human-associated species. Although bifidobacteria are traditionally classified as non-motile due to the absence of flagella^[51]^, the presence of a complete T4aP suggests the potential for surface-associated twitching motility in 12 taxa. Such a dynamic pilus system could provide enhanced surface sensing and niche exploration capabilities within host-associated environments^[52]^. These findings therefore point to a previously unrecognized level of functional diversification within the genus *Bifidobacterium*, challenging the traditional view of these bacteria as strictly non-motile and suggesting a potential contribution of T4aP to host adaptation.

### Investigating horizontal gene transfer events in T4P loci

To gain a comprehensive understanding of the evolutionary history of the identified T4P structures, a screening was performed to assess their genomic localization and occurrence of potential HGT events.

The HGT investigation was performed by analyzing CUB and GC content for each bifidobacterial genome, with thresholds dynamically calculated based on deviations from the values observed in the other coding sequences within the same genome. The analysis revealed that genes associated with the conserved T4cP structure were not acquired through HGT, whereas some genes associated with the species-specific T4aP structure appear to be putatively acquired. In particular, the* pil*X and* pil*E genes were the only ones with significant HGT signs (predicted to have been acquired over 30% of the time), with an even higher proportion (47%) observed for additional *pil*E genes placed outside the T4aP locus [Supplementary Table 5].

To investigate the localization of the conserved T4cP locus across bifidobacterial genomes, pangenome data were used to generate COGs, enabling the tracing of gene distribution. Analysis of the upstream and downstream genes flanking the locus revealed a high frequency of occurrence of similar COGs [Supplementary Table 6]. In 69% of the analyzed species, the upstream and downstream COGs were identical, indicating a conserved gene organization across genomes from different phylogenetic groups [Supplementary Table 6]. Less frequently distributed COGs were observed in a subset of phylogenetic groups, primarily those associated with the gut of primates, including the *B. bifidum*, *Bifidobacterium tissieri* (*B. tissieri*), and *B. longum* groups. These findings suggest that modifications in the genomic neighbourhoods of the T4cP locus mirror the evolutionary history of bifidobacteria, as the three most phylogenetically distant groups from the bifidobacterial ancestor display a conserved distribution of COGs in sub-clusters [Supplementary Table 6]. Interestingly, all bifidobacteria with this divergent locus configuration are primate-associated, which may reflect host-specific adaptations shaping the genomic context of the T4cP locus.

Overall, these results highlight that while the conserved T4cP locus is evolutionarily stable and vertically inherited within the *Bifidobacterium* genus, as supported by its consistent genomic context and lack of significant HGT signals. In contrast, while the core T4aP locus does not display strong evidence of recent horizontal acquisition, the detection of HGT signatures in specific genes, particularly *pil*X and accessory *pil*E copies located outside the main locus, suggests localized genetic remodelling rather than whole operon transfer. This pattern supports the view that T4aP represents a species-specific system that has been stably maintained within selected phylogenetic groups, with occasional gene-level exchanges potentially contributing to structural or functional diversification.

### Twitching motility of *B. adolescentis*

**Figure 4:**
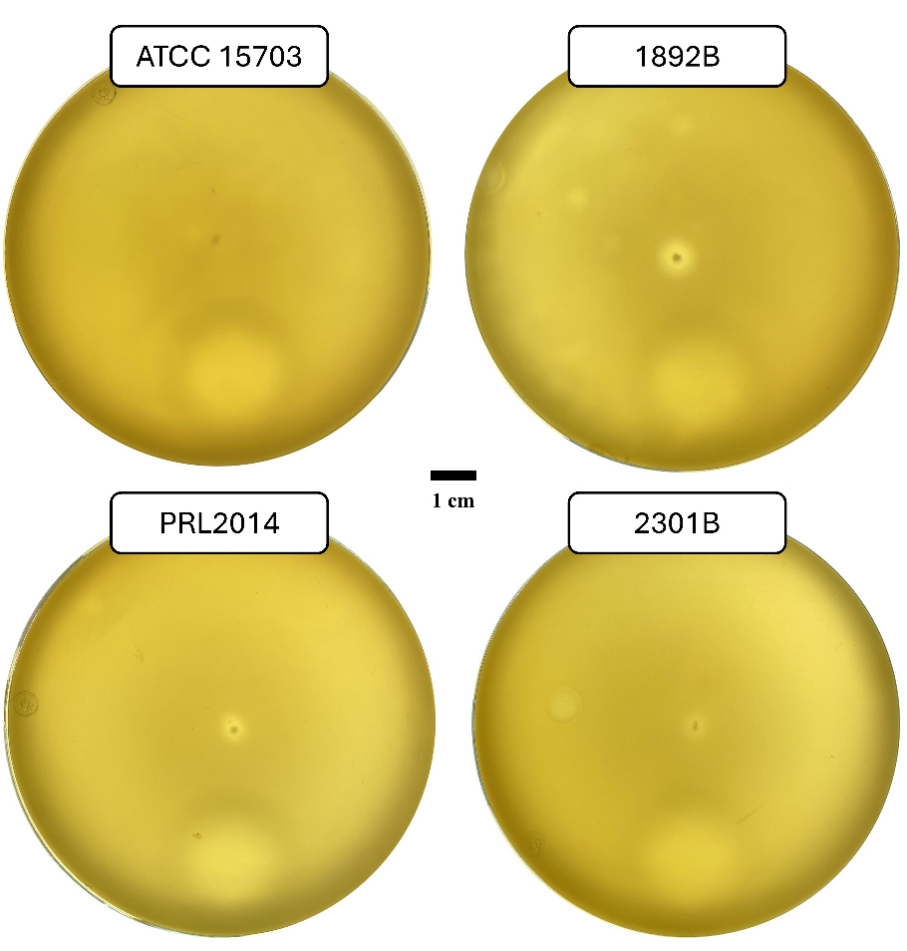
Macroscopic twitching assay of *B. adolescentis*. The image shows the formation of a growth halo surrounding the stab-inoculation site for all bifidobacterial strains whose T4aP locus was predicted through genomic investigation. Whereas no halo formation is observed at the stab-inoculation site of *B. adolescentis* ATCC 15703^T^, which lacks the locus and is therefore included as a negative control. Petri dish images were acquired using a Biobase colony counter, with plates placed above the instrument’s light source. The bar represents 1 cm. *B. adolescentis*: *Bifidobacterium adolescentis*; T4aP: type IVa pili; ATCC: American Type Culture Collection; *B. adolescentis*: *Bifidobacterium adolescentis*.

Twitching motility has been reported for several bacterial species and is commonly assessed using stab inoculation assays on semi-solid agar^[42,43]^. In this experimental setup, bacterial cells were stab-inoculated into the agar, resulting in the formation of an interstitial colony at the interface between the agar medium and the Petri dish^[42,53]^. Following incubation, the extent of the interstitial colony can be measured and used as an indicator of twitching motility. *B. adolescentis* strains 1892B, 2301B, and PRL2014, all predicted to encode the T4aP locus, were evaluated using this assay. As shown in Figure 4, these strains exhibited the formation of an interstitial halo when stab-inoculated onto 0.5% MRS agar supplemented with 2% starch. In contrast, *B. adolescentis* ATCC 15703^T^, used as a negative control lacking the T4aP locus, did not show detectable halo formation under the same conditions [Figure 4]. In addition, none of the bifidobacterial strains formed an interstitial halo when stab-inoculated onto 0.5% MRS agar in the absence of starch.

Overall, these observations suggest an association between the presence of the T4aP gene and the occurrence of twitching-like motility. However, the manifestation of this phenotype appears to be primarily influenced by the C source used. In this context, starch supplementation of the growth medium may act as a promoting factor, consistent with its role as a preferred carbon source for *B. adolescentis* strains^[41]^. These findings provide initial experimental support for the functional activity of the predicted T4aP locus and challenge the conventional classification of bifidobacteria as strictly non-motile organisms. Although further mechanistic investigations are required, the observed condition-dependent motility suggests that T4aP may contribute to environmental responsiveness and surface-associated behaviors.

### Study limitations

This study has some limitations that should be acknowledged. The phenotypic analyses were conducted on a limited number of strains, which may not fully capture the variability of T4aP-associated phenotypes. Although the selected *B. adolescentis* strains were chosen based on genomic predictions and the presence or absence of the T4aP locus, a broader strain panel would provide a more comprehensive assessment of the functional variability associated with this structure.

Experimental validation was restricted to *B. adolescentis* species. While this taxon is a biologically relevant and well-characterized member of the human gut microbiota, the T4aP locus has been identified in 12 additional bifidobacterial species. Therefore, extending phenotypic investigations to other species will be necessary to determine whether twitching motility and condition-dependent activation represent conserved traits within the genus.

The functional characterization of motility relied on agar-based twitching assays. Although this approach provided initial experimental evidence supporting T4aP-associated motility, more refined and quantitative methodologies will be required to dissect the mechanistic basis of this phenotype. In particular, high-resolution microscopic approaches, such as a microscopic twitching assay, as well as real-time imaging, would allow direct visualization of pilus dynamics and a more precise evaluation of motility behaviour.

### Conclusions

The comprehensive screening of T4P structures across the *Bifidobacterium* genus revealed a highly conserved T4cP (Tad) architecture present in all subspecies, with only minor structural variations within the *B. longum* and *B. boum* phylogenetic groups. The T4cP resembled an ancestral, non-retractile pilus typically associated with stable colonization and long-term host persistence. Alongside this conserved structure, a second and less common T4aP locus was identified in 13 bifidobacterial species. In contrast to the static nature of the T4cP, T4aP is predicted to be dynamic and retractile, suggesting a more adaptive role in environmental sensing and bacterial interactions. Notably, 12 of these species harbor a complete T4aP locus and cluster within phylogenetic groups associated with primate, bovine, and porcine hosts.

Using a twitching motility assay, we demonstrated that *B. adolescentis* strains exhibit motility in a condition-dependent manner, specifically upon starch supplementation and exclusively in strains harboring the T4aP locus. These findings provide experimental evidence supporting the functionality of T4aP and challenge the traditional view of bifidobacteria as strictly non-motile.

HGT analyses indicated that T4aP loci are largely conserved within species, indicating their stable integration in the genome. The identification of a functional T4aP in *B. adolescentis* and *B. pseudocatenulatum*, two species commonly associated with the human gut, highlights its potential role in host interaction and suggests a novel mechanism contributing to bifidobacterial adaptation within the human intestinal environment.

## DECLARATIONS

### Availability of data and materials

Genome sequences of all bifidobacteria included in this study are available in the public GenBank database of NCBI (https://www.ncbi.nlm.nih.gov/datasets/genome/) [Supplementary Table 3]. The manually curated bifidobacterial T4P database can be downloaded from the Computational Microbiology Unit (CMU) website at http://probiogenomics.unipr.it/cmu/. Additional scripts used in this project are available in the GitHub repository https://github.com/es95e/HGT_analysis.

### Copyright

© The Author(s) 2026.
